# The Reproducibility of Indoor Air Pollution (IAP) Measurement: A Test Case for the Measurement of Key Air Pollutants from the Pan Frying of Fish Samples

**DOI:** 10.1155/2014/236501

**Published:** 2014-06-25

**Authors:** Ki-Hyun Kim, Yong-Hyun Kim, Bo-Won Kim, Jeong-Hyeon Ahn, Min-Suk Bae, Richard J. C. Brown

**Affiliations:** ^1^Department of Civil & Environmental Engineering, Hanyang University, 222 Wangsimni-Ro, Seoul 133-791, Republic of Korea; ^2^Department of Environmental Engineering, Mokpo National University, Mokpo 534-729, Republic of Korea; ^3^Analytical Science Division, National Physical Laboratory, Teddington TW11 0LW, UK

## Abstract

To assess the robustness of various indoor air quality (IAQ) indices, we explored the possible role of reproducibility-induced variability in the measurements of different pollutants under similar sampling and emissions conditions. Polluted indoor conditions were generated by pan frying fish samples in a closed room. A total of 11 experiments were carried out to measure a list of key variables commonly used to represent indoor air pollution (IAP) indicators such as particulate matter (PM: PM_1_, PM_2.5_, PM_10_, and TSP) and a set of individual volatile organic compounds (VOCs) with some odor markers. The cooking activity conducted as part of our experiments was successful to consistently generate significant pollution levels (mean PM_10_: 7110 *μ*g m^−3^ and mean total VOC (TVOC): 1400 *μ*g m^−3^, resp.). Then, relative standard error (RSE) was computed to assess the reproducibility between different IAP paramters measured across the repeated experiments. If the results were evaluated by an arbitrary criterion of 10%, the patterns were divided into two data groups (e.g., <10% for benzene and some aldehydes and >10% for the remainders). Most noticeably, TVOC had the most repeatable results with a reproducibility (RSE) value of 3.2% (*n* = 11).

## 1. Introduction

The paradoxical aspect of cooking (that it is simultaneously necessary for human life but also exposes us to the risk of hazardous pollution) is an intriguing issue in environmental science. Cooking with fire generally releases many deleterious substances from the food and the fuels used for combustion. The types of pollutants released from these sources include particulate matter (PM) and the associated components (e.g., metals, polycyclic aromatic hydrocarbons (PAH), etc.) and gaseous constituents (e.g., carbon monoxide, formaldehyde, nitrogen dioxide, and volatile organic compounds) [1]. If these pollutants are not properly ventilated or treated, cooking activities can be considered the primary source of indoor air pollution (IAP) and pose significant threats not only to those who are cooking but also to those who reside in the same facility.

In light of the environmental significance of cooking activities and associated IAP, much research has been conducted to elucidate the effect of air pollutants released from cooking on human health. For instance, the associations between methods of cooking meats and colorectal cancer were assessed in a study performed in Stockholm in 1986–1988 [2]. If the significance of each variable (e.g., total meat intake, cooking style, etc.) was assessed based on the relative risks (RR) in relation to cancer type (colon versus rectum), the highest risks were found from fried meat with a heavily browned surface (RR colon = 2.8, RR rectum = 6.0). Similarly, dose-response relationships between cooking fume exposure and lung cancer were investigated among non-smoking Chinese women [3]. These authors relied on multiple unconditional logistic regressions to estimate the odds ratios (OR) for different levels of exposure, after adjusting for various potential confounding factors. They concluded that the risk of lung cancer in their target subjects was increased by cumulative exposure to any form of cooking by frying.

It is important to define indoor air pollutants currently of interest in order to inform the species to be measured by this study. For instance, a total of nine common indoor air pollutants (carbon dioxide, CO_2_; carbon monoxide, CO; respirable suspended particulates, RSP; nitrogen dioxide, NO_2_; ozone, O_3_; formaldehyde, HCHO; total volatile organic compounds, TVOC; radon, Rn; and airborne bacteria count, ABC) were officially selected as the parameters used in a certification scheme for office/public IAQ assessment in Hong Kong [4]. Often researchers rely on a reduced number of pollutants as markers for the full suite of possible indoor air pollutants. For instance, [4] the selected three (RSP, CO_2_, and TVOC) parameters based on such selection criteria include test sensitivity, specificity, and predictability.

The main objective of this study is to measure how reproducible pollutant concentration levels are, when the same source activity (i.e., pan frying of mackerel) is repeated at the same place but at different times. This gives some information about which IAP variables can be determined most consistently despite the presence of confounding sources of bias and variability (e.g., preparation of fish sample, cooking conditions, sampling, and analysis). The results of our study are thus expected to offer some insights into the most stable indoor air pollutants which could also be used as markers of pollution in assessing indoor environments.

## 2. Materials and Methods

### 2.1. Selection of Food Material and Target Components

In this work, we investigated a list of key pollutants that can be released from pan frying of fish, a common foodstuff in many countries. Indeed mackerel is one of the most common fish commercially available in Korean market places for its low price and unique taste favored by Koreans. Hence, we selected raw mackerel fish for pan frying. This has the additional benefit that its cooking can easily generate oil fumes and related pollutants.

To measure the pollutants released during pan frying, we focused on a list of volatile organic compounds (VOCs) that are well known as indoor pollutants and key offensive odorants such as acetaldehyde (AA), formaldehyde (FA), benzene (B), and TVOC [5]. Additionally, particulate matter in several size fractions (total suspended particulate (TSP), PM_1_, PM_2.5_, and PM_10_) and some odorous VOCs (*n* = 9) were also measured. The basic physicochemical properties (e.g., chemical formula, structural formula, molecular weight, CAS number, etc.) of all these compounds (*n* = 17) are summarized in Table 1. The basic information concerning sample collection and analysis of these target compounds is summarized in Table 2. In addition, detailed information of analytical procedures used for each air component is described in Table 2(d) along with operation conditions for all instrumental systems.

### 2.2. Sample Collection

**Figure 1:**
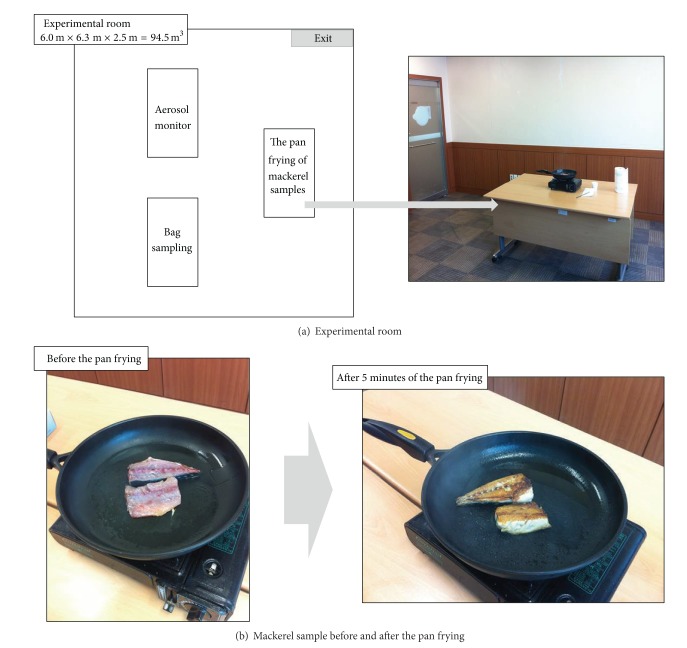
Photographs showing the mackerel samples investigated in this study.

For the purpose of this study, all mackerel samples were purchased fresh from a local market on the day of the experiment for the pan frying as discussed below. A total of 11 experiments were carried out over a three-day period in January 2014 (Table 2). All sampling was conducted in a room with capacity of 94.5 m^3^ (6.0 m × 6.3 m × 2.5 m (height)) (Figure 1). The temperature of the room ranged from 15 to 17°C during a sampling period over the three days. For each experiment, the new mackerel samples (approximately 200 g) were pan fired intensively for five minutes at the maximum flame setting on a portable grill using liquefied petroleum gas (LPG) without any additional oil or condiments. A significant amount of fumes and particulates appeared to be produced during these five minutes. The pan was placed on a desk (width: 0.6 m, length: 2 m, and height: 0.8 m). Sampling points were placed 1.5 m (vertically) and 2.5 m (horizontally) away from the pan.

The bag sampling and the measurement for PM data started immediately after turning off the portable grill (after 5 minutes of frying). Polluted air samples were then collected immediately into 10 L polyester aluminum (PEA) bags using a lung sampler (ACEN Co. Ltd., Korea). Upon collection of each sample, all windows and doors were opened to ventilate the polluted air before the next experiment. Because of this long ventilation requirement, the experiments were carried out at roughly three-hour intervals. The basic environmental conditions for each experiment are described in Table 2. All of the bag samples were then subjected to the GC-MS analysis for the target compounds. Concentrations of PM were measured in real time using aerosol monitor (Dusttrak DRX 8533, TSI understanding accelerated, USA) without the sampling steps.

### 2.3. Analysis of Target Compounds

The compounds chosen for analysis are those regularly referred to by indoor and ambient air legislation around the world as being harmful to human health or environmental sustainability or active as ozone precursors in the atmosphere.

#### 2.3.1. Formaldehyde and Acetaldehyde

The analysis of two aldehydes (formaldehyde (FA) and acetaldehyde (AA)) was carried out using high performance liquid chromatography (HPLC) equipped with a UV detector and dsCHROM software (for peak integration). The details of the HPLC system are summarized in Table 2. To undertake the analysis of the two aldehydes, samples in 10 L PEA bags were passed through Lp DNPH cartridges (Top Trading Co., Korea) for 5 min (at a fixed sampling flow rate of 1 L min^−1^) via a Sep-Pak ozone scrubber (Top Trading Co., Korea). Next, the target analytes caught up in the cartridges were eluted slowly with 5 mL acetonitrile and then filtered through 0.45 *μ*m, 13 mm, GHP Acrodisc filters (PALL, NY, USA) into a 25 mL borosilicate volumetric flask. The eluant was manually injected into the HPLC system, via a 20 *μ*L sample loop. The analytical precision, if assessed in terms of RSE, was 0.43% for FA and 0.18% for AA. The method detection limits (MDL) for FA and AA were 0.32 ppb and 0.28 ppb, respectively.

#### 2.3.2. VOC and TVOC Analysis

A TD system interfaced with GC and mass spectrometry (MS) was used for the analysis of VOCs ((1) aldehyde (*n* = 4), (2) ketone (*n* = 1), (3) aromatics (*n* = 4), and (4) ester (*n* = 1)) odorants, as listed in Table 1. The detailed operating conditions of this system are given in Table 2. The analysis of the VOCs was conducted by combining a gas chromatography system (GC: SHIMADZU GC-2010, Japan) equipped with a mass spectrometry system (MS: SHIMADZU GCMS-QP2010, Japan) and a thermal desorber (TD: Markes Ltd., UK). First, the gaseous samples loaded on the sorbent tube were thermally desorbed and passed through the cold trap unit of the TD at a preconcentration of 5°C. Then, the samples were thermally desorbed, transferred into the GC system, and separated on a CP-wax (60 m length, 0.25 mm diameter, and 0.25 *μ*m film thicknesses). The analysis of VOCs was performed in the following order: (1) transfer of samples loaded on the sorbent tube into the TD unit; (2) preconcentration on the cold trap by Carbopack C and B at a 1 : 1 volume ratio basis (Supelco, US) at 5°C; (3) thermal desorption at 330°C for 5 min; and (4) detection by the MS. The DL values for the VOCs ranged from 0.046 ppb (n-butyl acetate) to 0.253 ppb (propionic aldehyde).

The concentration of TVOC (*μ*g m^−3^) was estimated using the chromatograms obtained by the TD-GC-MS analysis. The TVOC values were calculated as the total concentrations of the target VOCs plus the nontarget VOCs found between n-hexane and n-hexadecane. The concentrations of nontarget VOCs were quantified using the response factor of toluene.

#### 2.3.3. PM Analysis

The PM released from mackerel samples by the pan frying was measured using aerosol monitor (Dusttrak DRX 8533, TSI understanding accelerated, USA). The mass concentrations of each PM fraction (PM_1_, PM_2.5_, PM_10_, and total PM) were estimated simultaneously using real-time 90° light-scattering laser photometers. The sampling flow rate for the measurement of PM was fixed at 3 L min^−1^. The quantification range of the aerosol monitor was 0.001 to 150 mg m^−3^.

## 3. Results and Discussion

### 3.1. The Level of IAP by Pan Frying Fish Samples

In this study, a total of 11 experiments were conducted to measure a set of indoor pollutants and odorant species released during cooking activities in a closed indoor environment. A summary of the measurements made is provided in Table 3.

For PM_10_ and TVOCs, high concentrations appeared to be generated consistently from each experiment with mean measured values of   7110 *μ*g m^−3^ and 1400 *μ*g m^−3^, respectively. For the reader's reference, these measurement data can be compared to the corresponding IAQ guidelines of 100 and 400 *μ*g m^−3^, respectively, which were set by the Korean Ministry of Environment [5]. The TVOC concentrations generated by fish frying appear to be several times higher than those observed from other source activities (e.g., the combustion of different BBQ charcoals) in our previous study [6]. In addition, a number of aldehyde species well known for offensive odorants such as acetaldehyde, valeraldehyde, and isovaleraldehyde were seen at the range over two orders of magnitude with mean concentrations of 99.3, 12.2, and 3.96 ppb, respectively. As such, these aldehyde species exceeded the guidelines set for malodor prevention law by the Korean Ministry of Environment [5].

**Figure 2:**
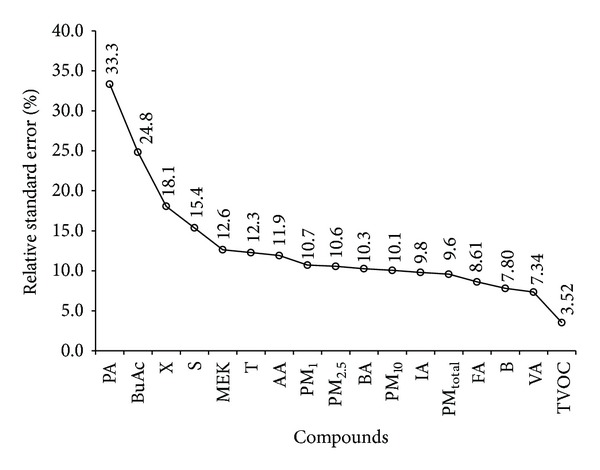
The relative standard error (RSE, %) values of the concentrations of target compounds obtained from a total of 11 repeat pan fryings of mackerel samples.

A principal component analysis (PCA) of the data collected (using OriginPro 9.1, OriginLab Corporation) demonstrated that the first two principal components accounted for 75% of the variability in the system. The first component was predominantly associated with aldehyde and TVOC content; the second competent was associated mainly with the aromatic component. However these relationships were not exclusive and most of the components showed some mutual correlation; the correlations determined between all components from the principal component analysis are shown in Table 4. Propionaldehyde stands out as having very low or negative correlations with most of the other components; this agrees well with observations of its poor reproducibility as seen in Figure 2. There is substantial correlation between the different PM measurements and also between the different aromatic components. TVOC also shows high correlation with many of the individual components, as would be expected since they are contributory components to the TVOC load.

**Figure 3:**
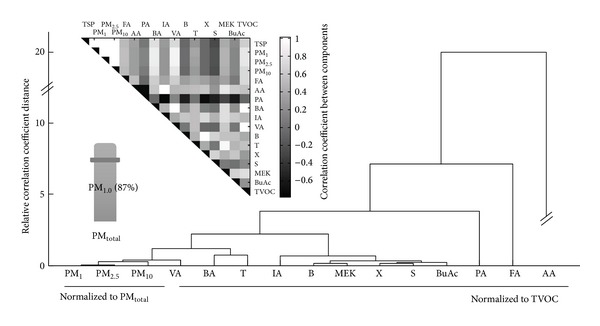
Hierarchical clustering analysis of the concentrations of target compounds obtained from a total of 11 repeat pan fryings of mackerel samples (note: PM_1_, PM_2.5_, and PM_10_ were normalized to PM_total_ and volatile organic compounds (VOCs) were normalized to TVOC).

Cluster analysis (CA) was performed additionally to evaluate the relationship between the measured PM and VOCs from pan frying fish samples in a closed room. Separation of variables into the two main clusters is plotted in Figure 3 as a dendrogram: (I) PM_1_, PM_2.5_, PM_10_, VA, BA, and T clusters; and (II) IA, B, MEK, X, S, and BuAc. These results are highly comparable to the patterns derived by the PCA discussed above as they identify correlations between the same pollutants groupings.

To learn more about the pollution induced by cooking, it is necessary to examine and evaluate chemical characteristics in PMs generated from pan frying fish samples. The results of multivariate statistical analysis (PCA and CA) confirm distinctly different associations among the studied PMs and VOCs; this suggests that parts of PMs could be formed by secondary aerosol production, possibly from VA, BA, and T, following the frying during the residence time of these compounds in air. Thus, we are planning to conduct future work in which the pollutants released from pan frying fish samples are quantified accurately in the atmospheric deposition collected in a closed room in the period after pan frying.

### 3.2. Representativeness of IAQ Parameters

In many countries, a list of common indoor air pollutants (e.g., CO_2_) is selected for IAQ assessment (e.g., [4]). Because of several issues (e.g., technical difficulties, such as maintenance of QA, cost, and limited resources), efforts have continuously been made to reduce or shorten the list of IAQ parameters while maintaining the representativeness of the measurement but identifying key marker components of IAQ. In this study, we attempted to examine the role of reproducibility as the performance index for such marker compounds which could be used in addition to common criteria (e.g., sensitivity, specificity, and predictability).

For the purpose of our study, we evaluated the reproducibility of each measure pollutant in terms of relative standard error (RSE, %). Figure 2 depicts the results of this comparison. The RSE values of most pollutant species were generally around 10% (e.g., <10% for benzene and some aldehydes and >10% for the other compounds). TVOC recorded the best reproducibility (RSE value of 3.2% (*n* = 11)). Note that the experimental uncertainties are controlled not only by the common variables in the analysis of airborne pollutants but also by all other variables involved in the sample treatment, preparation, and cooking procedures, especially the variation in the properties of the mackerel themselves (weight, surface area, and composition). However, this study only tests reproducibly under the very similar conditions to facilitate VOC emissions as described above. The results show that the measurement can be quite irreproducible, even under the constant conditions tested in the paper. It is true that changes in other conditions such as air flow would make a difference to the measured results, but these have not been tested here since it would not have been a simple task to deconvolve these contributions to the reproducibility post hoc, and further the reproducibility may then have been extremely large and may have undermined the usefulness of the study. However, it is important to acknowledge that under real conditions the uncertainty of such measurements would be increased further because of the variations in ventilation conditions at the cooking location and the size and geometry of the room.

These confounding factors, notwithstanding the reproducibilities found in this study, appear to be relatively consistent. TVOC would be a good candidate as a marker of IAP because it exhibited the most reproducible concentrations under field conditions. However, although a technical definition of TVOC has been proposed as sum of VOCs detected within the chromatographic VOC window [7], its reliability has been questioned due to the way in which different measurement systems realize this quantity [8] and the biases that this would therefore cause. Moreover, its representativeness has been challenged by a number of missing, but important, components such as low molecular weight reactive aldehydes (e.g., formaldehyde), intermediary reactants, and some volatile odorants at low threshold level [9–12]. Hence, it might be preferable to assign a specific subset of “marker” VOCs which are most likely to be stable and long-lived in air. Thus, their analysis should be well known and characterized in such a way that coherent SI traceable analytical measurements can be produced. Other compounds may undergo reactions in air at different rates depending on ambient conditions and therefore would not be suitable markers. When defining such marker compounds and threshold concentrations for IAQ, it is also important to standardize sampling conditions to ensure as far as possible consistency of the measurand.

## 4. Summary and Conclusions

In this study, a set of VOCs and offensive odorants released from the pan frying of fish samples was measured and their reproducibility was assessed using a total of 11 repeat experiments. Among all of the quantities evaluated, TVOC showed the lowest reproducibilities with an RSE value of 3.2%. The other pollutant species showed reproducibilities of around 10%. The results of our study suggest that TVOC can be used as highly meaningful marker of IAQ, although to ensure the SI traceability of the IAG parameters measured it may be preferable to select a few well-defined VOCs instead of TVOC.

## Summary of the Practical Implications

- The status of indoor air pollution (IAP) is an important factor to assess its health impact on the residents.
- The reproducibility between IAP variables is assessed critically by a series of repetitive experiments.
- The relative importance between IAP variables is clearly distinguished when measured repeatedly.

## Figures and Tables

**Table:** (a) Gaseous VOCs

Order	Group	Compounds	Short name	MW (g mol^−1^)	Density (g cm^−3^)	Boiling point (°C)	Formula	CAS number
1	Aldehyde	Formaldehyde	FA	30.03	0.8153	−19	CH_2_O	50-00-0
2		Acetaldehyde	AA	44.1	0.785	20.2	C_2_H_4_O	75-07-0
3		Propionaldehyde	PA	58.1	0.798	46-50	C_3_H_6_O	123-38-6
4		Butyraldehyde	BA	72.1	0.805	74.8	C_4_H_8_O	123-72-8
5		Isovaleraldehyde	IA	86.1	0.797	90-93	C_5_H_10_O	590-86-3
6		n-Valeraldehyde	VA	86.1	0.81	102-103	C_5_H_10_O	110-62-3

7	Aromatic	Benzene	B	78.11	0.878	80.1	C_6_H_6_	71-43-2
8		Toluene	T	92.14	0.866	111	C_7_H_8_	108-88-3
9-1		p-Xylene	p-X	106.2	0.865	138	C_8_H_10_	106-42-3
9-2		m-Xylene	m-X	106.2	0.865	139	C_8_H_10_	108-38-3
9-3		o-Xylene	o-X	106.2	0.88	144	C_8_H_10_	95-47-6
10		Styrene	S	104.2	0.906	145	C_8_H_8_	100-42-5

11	Ketone	Methyl ethyl ketone	MEK	72.11	0.805	79.64	C_4_H_8_O	78-93-3
12	Ester	n-Butyl acetate	BuAc	116.2	0.881	126	C_6_H_12_O_2_	123-86-4

**Table:** (b) Others

Order	Name	Short name
1	Total suspended particulate	PM_total_
2	Particulate matter 1	PM_1_
3	Particulate matter 2.5	PM_2.5_
4	Particulate matter 10	PM_10_

5	Total volatile organic compounds	TVOC

**Table:** (a) Information of mackerel sample

Order	Sample code	Weight (g)	Room Temperature (°C)	Sampling	
				Date	Time
1	M1	192	15.3	27 Jan. 2014	10:03
2	M2	169	17.0	27 Jan. 2014	13:10
3	M3	200	16.5	27 Jan. 2014	16:11
4	M4	194	16.1	27 Jan. 2014	19:03

5	M5	186	16.2	28 Jan. 2014	10:20
6	M6	167	16.9	28 Jan. 2014	13:31
7	M7	184	16.4	28 Jan. 2014	16:07
8	M8	189	15.8	28 Jan. 2014	19:09

9	M9	189	17.1	29 Jan. 2014	11:08
10	M10	191	16.5	29 Jan. 2014	14:10
11	M11	195	15.9	29 Jan. 2014	17:07

**Table:** (b) Information of sampling

(1) Target compounds:	Gaseous VOCs (*n* = 12)
(2) Sampling approach:	Lung sampling
(3) Sample container:	10 L polyester aluminum (PEA) bag
(4) Sampling flow rate:	20 L min^−1^
(5) Sampling time:	0.5 min
(6) Sample volume:	10 L

**Table:** (c) Analytical method

Order	Target compounds	Pretreatment	Separation system	Detector
1	Particulate matters (*n* = 4)	—	—	90° light-scattering laser photometer

2	FA	DNPH-cartridge	HPLC: Acclaim 120 C18	UV
	AA			

3	Aldehyde (4)	Sorbent tube/thermal desorption	GC: wax column (30 m × 0.25 mm × 0.25 *μ*m)	Quadrupole mass spectrometry
	Aromatics (4)			
	Ketone (1)			
	Ester (1)			

**Table:** (d) Operational description of two instrumental systems (HPLC and GC) in this study


(A) HPLC/UV (Spectrasystem UV2000, Thermo scientific, USA) system for carbonyl compounds analysis			
(i) Injector		(iii) Detector (UV)	
Volume:	20 *μ*L	Wavelength:	360 nm
(ii) Pump		(iv) Column (C_18_, Hichrom, UK)	
Flow rate:	1.5 mL/min	Length:	250 mm
Mobile phase:	70 : 30 acetonitrile : H_2_O	Diameter:	4.6 mm
Analysis time:	16 min	Particle size:	5 *μ*m

(B) TD-GC/MS system			
(1) GC (SHIMADZU GC-2010, JAPAN) and MS (SHIMADZU GCMS-QP2010, JAPAN) system			
(i) Oven		(ii) Detector (MS)	
1st oven temperature:	40°C (5 min.)	Ionization mode:	EI (70 eV)
1st oven rate:	5°C min^−1^	Ion source temperature:	230°C
2nd oven temperature:	220°C (5 min.)	Interface temperature:	230°C
			
Total time:	46 min	TIC scan range:	35~600 M z^−1^
Carrier gas:	He (99.999%)		
Column flow:	1 mL min^−1^		
(2) Thermal desorber (UNITY II, Markes International, Ltd., UK) condition			
Cold trap:	Carbopack C + Carbopack B (volume ratio = 1 : 1)		
Split ratio:	1 : 5	Trap temp (low):	5°C
Split flow:	5 mL min^−1^	Trap temp (high):	330°C
Trap hold time:	5 min	Flow path temperature:	180°C

**Table 3:** Concentrations of PM and gaseous VOCs released from mackerel samples by the pan frying.

Order	Target compounds	Unit	Sample code													
			M1	M2	M3	M4	M5	M6	M7	M8	M9	M10	M11	Mean	SD	RSE
(a) Particulates																
1	PM_total_	mg/m^3^	4.51	9.40	8.24	8.98	3.38	6.29	6.53	12.1	7.07	7.84	7.76	7.46	2.37	9.57
2	PM_1_	mg/m^3^	3.79	8.64	5.94	7.39	2.82	5.02	5.74	11.3	6.58	7.73	7.24	6.56	2.33	10.7
3	PM_2.5_	mg/m^3^	3.88	8.71	6.15	7.60	2.86	5.15	5.87	11.4	6.68	7.76	7.29	6.67	2.33	10.6
4	PM_10_	mg/m^3^	**4.26**	**9.10**	**7.24**	**8.27**	**3.09**	**5.67**	**6.28**	**11.9**	**6.88**	**7.82**	**7.66**	**7.11**	2.37	10.1

(b) Gaseous VOCs																
5	FA	ppb	39.4	33.4	24.2	39.8	18.1	42.0	40.3	53.0	38.1	44.8	23.9	36.1	10.3	8.61
6	AA	ppb	**105**	**138**	**52.7**	**144**	**83.9**	**153**	**73.9**	**144**	**54.1**	**85.7**	**58.2**	**99.3**	39.2	11.9
7	PA	ppb	1.23	1.84	16.8	1.74	1.55	1.84	19.2	1.63	27.0	40.6	30.9	13.1	14.5	33.3
8	BA	ppb	5.67	8.62	3.90	10.8	4.63	8.45	5.39	12.5	8.22	8.87	8.84	7.81	2.66	10.3
9	IA	ppb	**4.67**	**5.43**	2.57	**4.89**	**4.16**	**5.08**	2.74	**5.74**	2.03	**3.33**	2.93	**3.96**	1.29	9.8
10	VA	ppb	8.94	**12.9**	7.73	**13.6**	8.88	**13.3**	**10.1**	**17.6**	**12.5**	**14.7**	**13.9**	**12.2**	2.97	7.34
11	B	ppb	3.39	2.38	1.56	2.51	1.79	2.01	1.48	2.28	1.94	1.80	1.82	2.09	0.54	7.80
12	T	ppb	10.3	12.9	3.4	10.4	6.26	8.82	5.10	9.13	4.90	14.9	8.64	8.62	3.51	12.3
13	X	ppb	2.88	1.86	0.56	1.31	0.93	1.29	0.91	1.64	0.54	0.67	0.56	1.20	0.72	18.1
14	S	ppb	2.16	0.75	0.62	0.79	1.62	0.99	0.57	1.75	1.03	0.70	0.65	1.06	0.54	15.4
15	MEK	ppb	2.09	3.09	0.76	3.12	1.38	2.30	0.74	2.35	1.31	2.39	2.06	1.96	0.82	12.6
16	BuAc	ppb	0.54	1.16	0.15	0.71	0.25	0.41	0.24	0.24	0.18	0.15	0.17	0.38	0.31	24.8
17	TVOC	*µ*g/m^3^	**1,375**	**1,452**	**1,115**	**1,554**	**1,204**	**1,390**	**1,261**	**1,703**	**1,440**	**1,471**	**1,439**	**1,400**	163	3.52

Bold-phased values denote those exceeding the emission guideline levels designated by the indoor pollution regulation guideline (in case of PM) or the malodor prevention law of Korea [5].

**Table 4:** Correlation matrix resulting from the principal component analysis of the data produced in this study.

	PM_1_	PM_2.5_	PM_10_	FA	AA	PA	BA	IA	VA	B	T	X	S	MEK	BuAc	TVOC
TSP	0.97	0.97	0.99	0.50	0.34	0.03	0.74	0.28	0.71	−0.03	0.25	−0.05	−0.27	0.41	0.17	0.66
PM_1_	—	1.00	0.99	0.55	0.32	0.13	0.80	0.27	0.82	−0.02	0.37	−0.04	−0.21	0.46	0.14	0.76
PM_2.5_		—	0.99	0.55	0.32	0.12	0.80	0.27	0.81	−0.02	0.36	−0.04	−0.22	0.45	0.15	0.76
PM_10_			—	0.52	0.33	0.08	0.77	0.27	0.76	−0.03	0.30	−0.04	−0.24	0.42	0.15	0.71
FA				—	0.54	−0.05	0.64	0.37	0.64	0.31	0.41	0.34	0.18	0.36	0.06	0.71
AA					—	−0.71	0.59	0.94	0.48	0.51	0.54	0.60	0.27	0.76	0.63	0.58
PA						—	−0.05	−0.78	0.13	−0.53	0.02	−0.68	−0.54	−0.30	−0.58	−0.08
BA							—	0.49	0.96	0.23	0.53	0.10	0.01	0.73	0.21	0.97
IA								—	0.39	0.60	0.54	0.71	0.47	0.74	0.63	0.52
VA									—	0.05	0.54	−0.03	−0.06	0.63	0.05	0.92
B										—	0.45	0.91	0.67	0.57	0.56	0.41
T											—	0.40	0.05	0.85	0.49	0.58
X												—	0.68	0.46	0.61	0.28
S													—	0.08	0.01	0.19
MEK														—	0.68	0.73
BuAc															—	0.24
TVOC																—
